# Development of Glomerulus-, Tubule-, and Collecting Duct-Specific mRNA Assay in Human Urinary Exosomes and Microvesicles

**DOI:** 10.1371/journal.pone.0109074

**Published:** 2014-10-02

**Authors:** Taku Murakami, Melanie Oakes, Mieko Ogura, Vivian Tovar, Cindy Yamamoto, Masato Mitsuhashi

**Affiliations:** 1 Hitachi Chemical Research Center, Inc., Irvine, California, United States of America; 2 Genomics High-Throughput Facility, University of California Irvine, Irvine, California, United States of America; 3 NanoSomiX, Inc., Irvine, California, United States of America; University of Geneva, Switzerland

## Abstract

Urinary exosomes and microvesicles (EMV) are promising biomarkers for renal diseases. Although the density of EMV is very low in urine, large quantity of urine can be easily obtained. In order to analyze urinary EMV mRNA, a unique filter device to adsorb urinary EMV from 10 mL urine was developed, which is far more convenient than the standard ultracentrifugation protocol. The filter part of the device is detachable and aligned to a 96-well microplate format, therefore multiple samples can be processed simultaneously in a high throughput manner following the isolation step. For EMV mRNA quantification, the EMV on the filter is lysed directly by adding lysis buffer and transferred to an oligo(dT)-immobilized microplate for mRNA isolation followed by cDNA synthesis and real-time PCR. Under the optimized assay condition, our method provided comparable or even superior results to the standard ultracentrifugation method in terms of mRNA assay sensitivity, linearity, intra-assay reproducibility, and ease of use. The assay system was applied to quantification of kidney-specific mRNAs such as *NPHN* and *PDCN* (glomerular filtration), *SLC12A1* (tubular absorption), *UMOD* and *ALB* (tubular secretion), and *AQP2* (collecting duct water absorption). 12-hour urine samples were collected from four healthy subjects for two weeks, and day-to-day and individual-to-individual variations were investigated. Kidney-specific genes as well as control genes (*GAPDH*, *ACTB*, etc.) were successfully detected and confirmed their stable expressions through the two-week study period. In conclusion, this method is readily available to clinical studies of kidney diseases.

## Introduction

Kidney failure is one of the growing public health issues causing economic and social impacts. The number of chronic kidney disease patients is more than twenty million in the United States alone [1]. Kidney failure will eventually progress to high cost medical care such as dialysis and kidney transplantation. Patients also suffer from not only kidney failure but also other complications such as increased incidents of cardiovascular disease, hyperlipidemia, anemia, and mineral and metabolic bone disorder [1]. Thus, the early detection, prevention, and management of kidney disease are huge unmet needs in medicine.

The stages of kidney disease are mainly diagnosed by estimated GFR (eGFR) by an empirical formula using serum creatinine concentration, age, sex, and race. Blood urea nitrogen and microalbuminuria are also established biomarkers for kidney disease progression. However, since these biomarkers are not sensitive enough to predict kidney disease progression, various new biomarkers have been proposed recently such as neutrophil gelatinase-associated lipocalin (NGAL), kidney injury molecule-1 (KIM-1) and liver-type fatty acid-binding protein (L-FABP), and are under thorough clinical validations [2], [3].

Urinary exosomes and microvesicles (EMV) have been investigated extensively as promising biomarkers in the recent years. Exosomes are 30–100 nm vesicles released from cells through fusion of multivesicular bodies to the plasma membranes and found in most of human body fluids such as blood, urine, cerebrospinal fluid and saliva [4], [5]. Microvesicles are 100–1000 nm vesicles also released from cells however directly from the plasma membranes [4]. Both vesicles encapsulate intracellular proteins, mRNA and microRNA that are released from epithelial cells of the nephron into urine. Therefore, molecular analysis of urinary EMV may elucidate the kidney functions at molecular levels.

Urine has been a valuable biofluid for diagnostics since it is easily obtained in large quantities without any harm to the patients. However, urine comprises of chemically complex components and may contain red blood cell, white blood cell, epithelial cell, cast, bacteria, virus, protein, metabolites, salt, etc. in addition to EMV. Unlike blood or other biological fluids, biochemical parameters of urine such as pH and the concentration of salt vary greatly among subjects, health conditions, and sample collection procedures. The most prevalent standard method to isolate EMV from human urine is ultracentrifugation [6], [7]. It is efficient and useful for research purposes, however may not be practical for routine use at clinical laboratories because the protocol is too lengthy, tedious, and low throughput. There are several other methods available to isolate urinary EMV such as immunomagnetic beads, nanomembrane filtration [8] and polymer-based co-precipitation [9], however their recovery yield and purity vary greatly among the methods [6], [10], [11], and none are used as clinical diagnostics yet. In order to use urinary EMV for routine clinical diagnostics, a new method is needed.

Recently, we developed a unique 96-well filterplate in order to isolate EMV from human plasma samples for mRNA analysis [12]. Although we were able to detect EMV mRNA in human urine using the same system, it was necessary to process 5–12 mL urine to obtain sufficient sensitivity. A standard 96-well filterplate format is useful for high-throughput assays, but not convenient to process samples with >1 mL sample volumes. On the other hands, a standard centrifuge filter tube format is useful to process large volumes of samples, but not suitable for high-throughput assays. In this study, we developed a unique EMV mRNA quantification method from 10 mL urine samples in a high throughput format, and quantified kidney-specific mRNAs. Analytical validation has been completed, and the system is ready for clinical research, biomarker screening, and the development of molecular diagnostics.

## Materials and Methods

### Materials

PCR primers were obtained from Integrated DNA Technologies (Coralville, IA) (sequences available in Table S1). MMLV reverse transcriptase and RNasin were purchased from Promega (Madison, WI) and SsoAdvanced SYBR Green Supermix was from Bio-Rad (Hercules, CA). Anonymous human urine samples were obtained from healthy volunteers following the guideline from the Office for Human Research Protections (OHRP) [13]. This study was approved and the informed consent requirement was waived by the ethical committee of Hitachi Chemical Research Center, Inc. Samples were anonymously delivered to a designated box by the volunteers. Spot urine samples were stored at 4°C up to 4 hours and at −80°C for longer storage. 12-hour urine samples were obtained by pooling urine between 8 pm and 8 am next day including the first morning urine. The samples were stored in a Styrofoam box with chemical ice packs during the sampling and stored at −80°C after the last sampling.

### Scanning Electron Microscope (SEM) Analysis of Urinary EMV

A spot urine sample was centrifuged at 800×g for 15 min to remove large particles. The supernatant was applied to exosome collection tube (Hitachi Chemical Research Center (HCR), Irvine, CA) and centrifuged at 2,000×g for 10 min. The filter was fixed with 4% paraformaldehyde in 1× Phosphate buffer saline, pH 7.4 (PBS) for 5 min, rinsed with 0.05M glycine PBS and 1% casein PBS each with 15-min incubation. The filter was stained with anti-human CD63 antibody (Biolegend, San Diego, CA) at 5 µg/mL in 1% casein PBS for 60 min. After three washes with 1% casein PBS, the filter was stained with 10-nm gold colloid labeled anti-IgG antibody (Sigma-Aldrich, St. Louis, MO) at 1/40 dilution in 1% casein PBS for 2 hours and rinsed three times each with 1% casein PBS and PBS with 3-min incubation. After the second fixation with 4% paraformaldehyde in PBS for 5 min, the filter was rinsed once with PBS and twice with distilled water with 3-min incubation. The conjugated gold particles on the filter were treated with silver enhancement reagent (SPI Supplies, West Chester, PA) for 15 min. After five washes with distilled water, the filter was dried overnight and analyzed by SEM (S-4800, Hitachi High-Technologies, Tokyo, Japan).

### Urinary EMV Isolation by Differential Centrifugation

Urinary EMV were isolated from human urine using differential centrifugation. Urine samples were centrifuged at 3,000×g for 10 min to remove cellular debris. The supernatants were collected and centrifuged at 3,000×g for 10 min, again. The supernatants were further centrifuged at 10,000×g for 30 min to remove large particles, and the supernatants were collected. EMV in the supernatants were precipitated by ultracentrifugation (100,000×g for 1 hour), rinsed with PBS, then precipitated and suspended in 1× PBS. Nanoparticle tracking analysis of the obtained urinary EMV was conducted by Nanosight LM20 (Nanosight, Novato, CA).

### EMV mRNA Analysis from Urine Samples

Human urine samples were processed as follows unless otherwise noted. Urine sample were centrifuged at 800×g for 15 min to remove large particles. The supernatants were collected carefully and mixed with 1/4 volumes of 25× PBS, pH 7.4. 12.5 mL of the mixtures (10 mL urine supernatants) were applied to exosome collection tubes (HCR) and centrifuged at 2,000×g for 10 min. Eighty µL of Lysis buffer [14] were added to the filters, and incubated at 37°C for 10 min. The lysates were then transferred to an oligo(dT)-immobilized microplate (HCR) by centrifugation and incubated at 4°C for overnight for mRNA hybridization [14]. After six washes with Wash buffers, cDNA was synthesized in the same microplate by adding 30 µL of 1× reverse transcription buffer containing 1.25 mM each of dNTPs, 2.7 U/µL MMLV reverse transcriptase and 0.13 U/µL RNasin, and incubated at 37°C for 2 hours. Real-time PCR was conducted using ABI 7900HT or ViiA7 real-time PCR system (Life Technologies, Carlsbad, CA) in a 5 µL reaction containing 1× SsoAdvanced SYBR Green Supermix and 500 nM each of primer pairs. Analyzed mRNAs and their primer sequences are available in Table S1. The temperature profile was 40 cycles of 95°C for 30 sec and 65°C for 1 min after the initial denaturation at 95°C for 10 min, followed by melting curve analysis. Real-time PCR data was analyzed by the instrument control software and Microsoft Excel. Gene copy number per sample was obtained by converting threshold cycle values to gene copy numbers using reference curves and an estimated mRNA recovery rate, 10%, by the oligo(dT) microplate [14].

### EMV mRNA analysis by the Standard Ultracentrifugation Method

Urine samples were centrifuged at 800×g for 15 min to remove large particles. The supernatants were collected carefully and centrifuged at 100,000×g for 1 hour at 4°C to precipitate EMV. The pellets were obtained by decantation of the supernatants. The pellets were lysed in 80 µL of Lysis buffer containing a cocktail of antisense primers at 37°C for 10 min. The lysates were transferred to an oligo(dT)-immobilized microplate by pipetting and incubated for mRNA hybridization, followed by cDNA synthesis and real-time PCR as described above.

## Results

### Filter Device to Isolate Exosomes and Microvesicles from Human Urine

**Figure 1:**
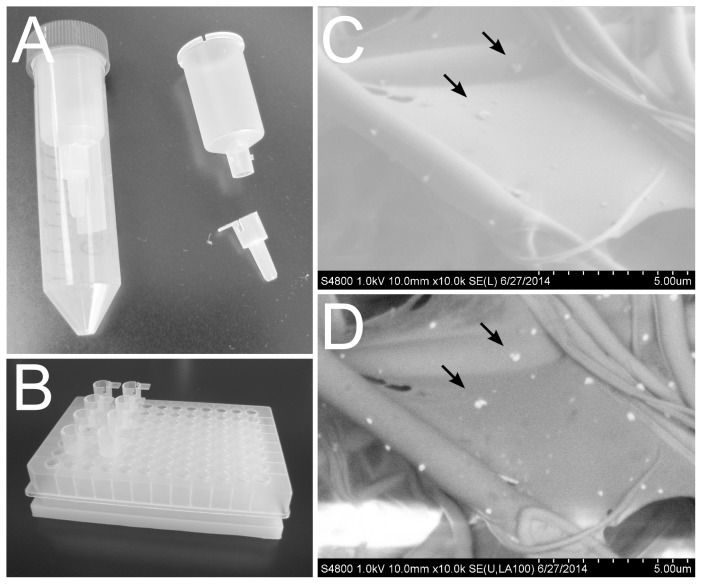
Exosomes and Microvesicles (EMV) Capture Device. A. A picture of assembled EMV capture device (left) and its components: syringe tube (top right) and filter tip (bottom right). B. A picture of filter tips aligned in a 96-well microplate format on top of oligo(dT)-immobilized microplate. C, D. Scanning electron microscope (SEM) analysis of urinary particles captured on the filter fiber. The filter was labeled with anti-CD63 antibody and colloidal gold with silver enhancement. The filter was analyzed by the lower detector mode (C) and the backscattering mode (D).

In order to process large volumes of urine samples without compromising the assay throughput during the downstream mRNA quantification process, a unique EMV capture device was developed (Figure 1A). The device can filter 10-mL urine sample by 10-min low speed centrifugation. By repeating this process a couple of times, 20–30 mL urine can be analyzed without clogging. Once a sample is filtered, the filter tip is detachable and aligned to a 96-well microplate format (Figure 1B). Therefore, multiple samples can be processed simultaneously in a high throughput manner following the EMV isolation. Interestingly, the filter tip is so small that only a limited volume of lysis buffer is sufficient to wet the entire filter membrane. Thus, this device is a unique interface between mL and µL solutions. Moreover, the filtrate is enclosed in a 50 mL centrifugation tube, and is discarded safely, allowing us to use biohazardous samples with ease. For EMV mRNA quantification, the EMV on the filter is lysed directly by adding lysis buffer and the EMV lysate is transferred to an oligo(dT)-immobilized microplate for mRNA isolation followed by cDNA synthesis and real-time PCR as previously described [12], [14], [15].

When human urine was applied to the filter, urinary particles were adsorbed on the filter fibers (Figure S1). These urinary particles may be EMV as the particle diameters ranged from 68 to 232 nm. In order to investigate if the bound urinary particles have exosome surface markers, immunogold labeling of the filter was conducted using anti-CD63 and anti-CD9 antibodies. Following silver enhancement of the gold labeling, the filter turned brown, which indicates the presence of these exosome surface markers on the filter, while the filter without primary antibody did not (data not shown). The SEM analysis of the filter labeled with anti-CD63 antibody confirmed less than 200-nm particles on the filter fibers by the lower detector mode (Figure 1C) and bright spots due to gold and silver at the same locations by the backscattering mode (Figure 1D), indicating that these particles are the silver enhanced gold colloids around the urinary particles. Furthermore, as indicated by the arrows, a few particles were aggregated together in close proximity suggesting that multiple gold colloids were attached to the same urinary particles simultaneously. Similar results were obtained in the filter sample stained with anti-CD9 antibody (data not shown). Therefore, it was confirmed that the urinary particles captured on the filter were bona fide exosomes.

**Figure 2:**
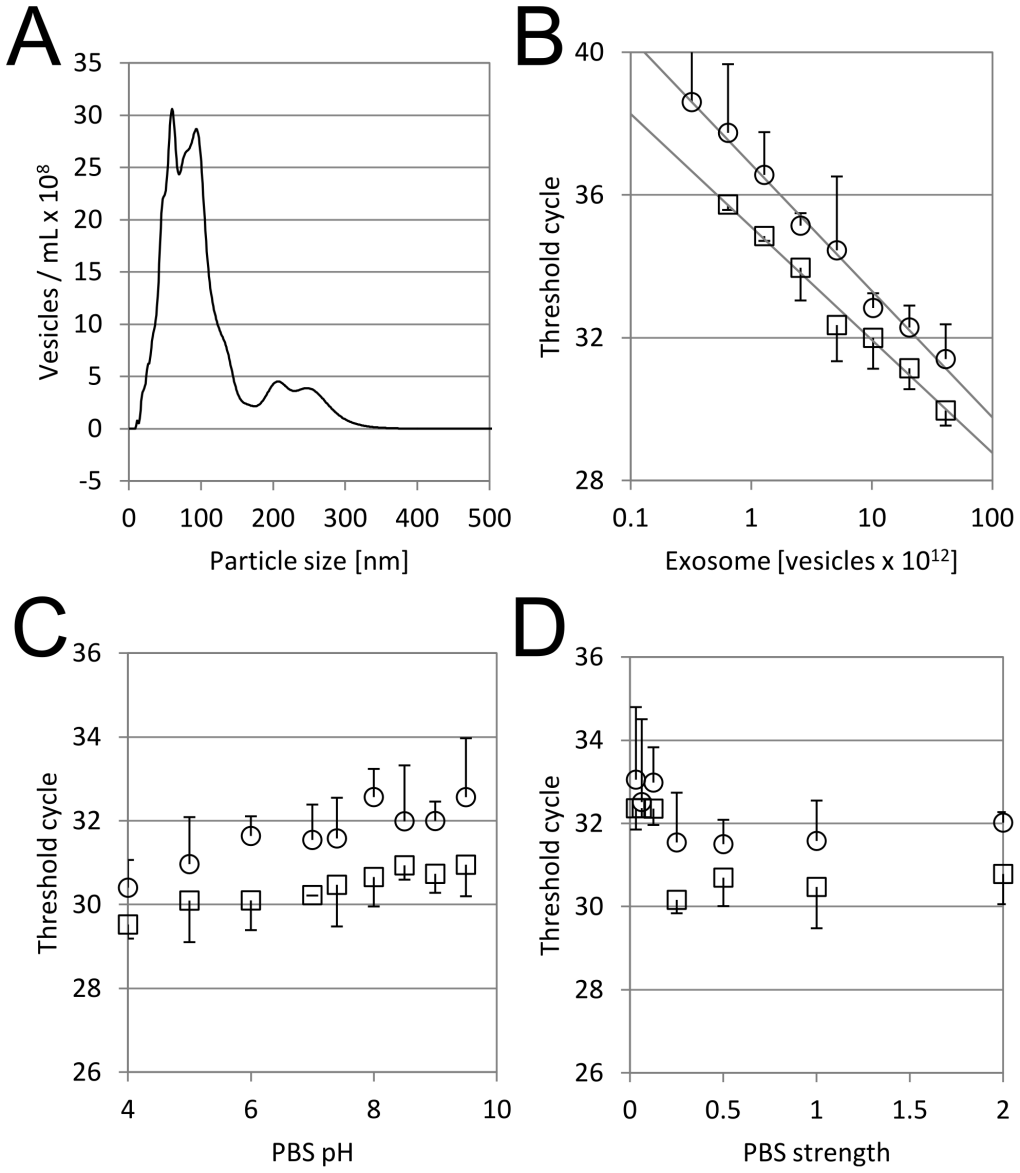
Performance Characterization using Isolated Urinary EMV. A. Urinary EMV were isolated from human urine using a differential centrifugation method and analyzed by nanoparticle tracking analysis as described in Materials and Methods. B–D. Urinary EMV was diluted in phosphate buffer saline (PBS) with different EMV concentrations (B), pH (C) or buffer strength (D), then applied to the filter material. *ACTB* (○) and *GAPDH* (□) were quantified in triplicate as described in Materials and Methods. Mean Ct values were plotted and error bars are standard deviations.

In order to further characterize the filter material, urinary exosome was isolated from human urine by a differential centrifugation method. Nanoparticle tracking analysis confirmed that the majority of isolated vesicles were less than 100 nm in diameter, which is consistent with published size ranges of exosomes (Figure 2A) [4]. Additional broad peaks between 200 and 300 nm were also observed, which may correspond to microvesicles. It was difficult to distinguish vesicles corresponding to the additional peaks from exosomes because the exosomes outnumbered the larger vesicles (Figure 2A, S1). Using the purified EMV, it was confirmed that the filter material could capture EMV in a dose dependent manner (Figure 2B) and more than 99% of the applied EMV were captured (Figure S2). EMV was successfully captured in a wide range of pH between pH 4 and 9.5 and lower pH showed slightly better capture efficiency than higher pH (Figure 2C). EMV capture efficiency was consistent in a wide range of salt concentrations between 0.25× and 2× PBS, however it was less efficient at less than 0.25× PBS (Figure 2D).

### Urinary EMV mRNA Assay

**Figure 3:**
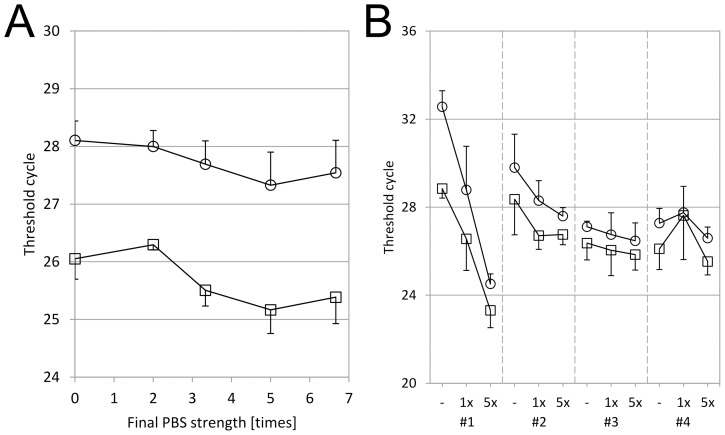
pH and Salt Concentration Adjustment for Efficient EMV Capture. A. Spot urine from a healthy donor was centrifuged at 800×g for 15 min and the supernatant was collected. Two mL of the supernatant was mixed with 0, 0.5, 1, 2 or 4 mL of 10× PBS, then applied to EMV capture devices. *ACTB* (○) and *GAPDH* (□) were quantified in triplicate as described in Materials and Methods. Mean Ct values were plotted and error bars are standard deviations. B. Twelve-hour urine samples were obtained from four healthy donors (#1 to #4). The samples were centrifuged at 800×g for 15 min and the supernatants were collected. Four and a half mL of the supernatants were mixed with 0 mL (-), 0.5 mL (1×) or 4.5 mL (5×) of 10× PBS, then applied to EMV capture devices. *GAPDH* (○) and *RPLP0* (□) were quantified in triplicate as described in Materials and Methods. Mean Ct values were plotted and error bars are standard deviations.

When this device was applied to human urine samples, we found that some samples did not work well for EMV isolation, probably due to the difference in pH or salt concentration. As mentioned previously, these parameters may vary among subjects and health conditions, thus pH and salt concentration should be adjusted prior to sample filtration. To simplify this process, we added concentrated PBS in urine samples. First, the final strength of PBS was optimized. Zero to two volumes of 10× PBS were mixed with urine samples prior to filtration, and two control genes were quantified. Threshold cycle (Ct) values decreased as the strength of PBS increased, and the lowest Ct or maximum sensitivity was achieved when more than 5× PBS at final buffer strength was added (Figure 3A). Similar results were obtained using the other buffers such as HEPES/NaCl buffer (data not shown). In order to investigate individual difference on the effect of PBS adjustment, zero to one volume of 10× PBS was added to 12-hour urine samples from four individuals, and two control genes were quantified (Figure 2B). The addition of PBS improved the assay sensitivity greatly for Subject #1 by Ct differences of more than 8 for *GAPDH* and 5 for *RPLP0.* For Subjects #2–#4, the PBS addition showed no negative effects. These data suggest that the sample variation of pH and salt concentrations can be normalized by adding a concentrated buffer solution to make mRNA assay results consistent.

**Figure 4:**
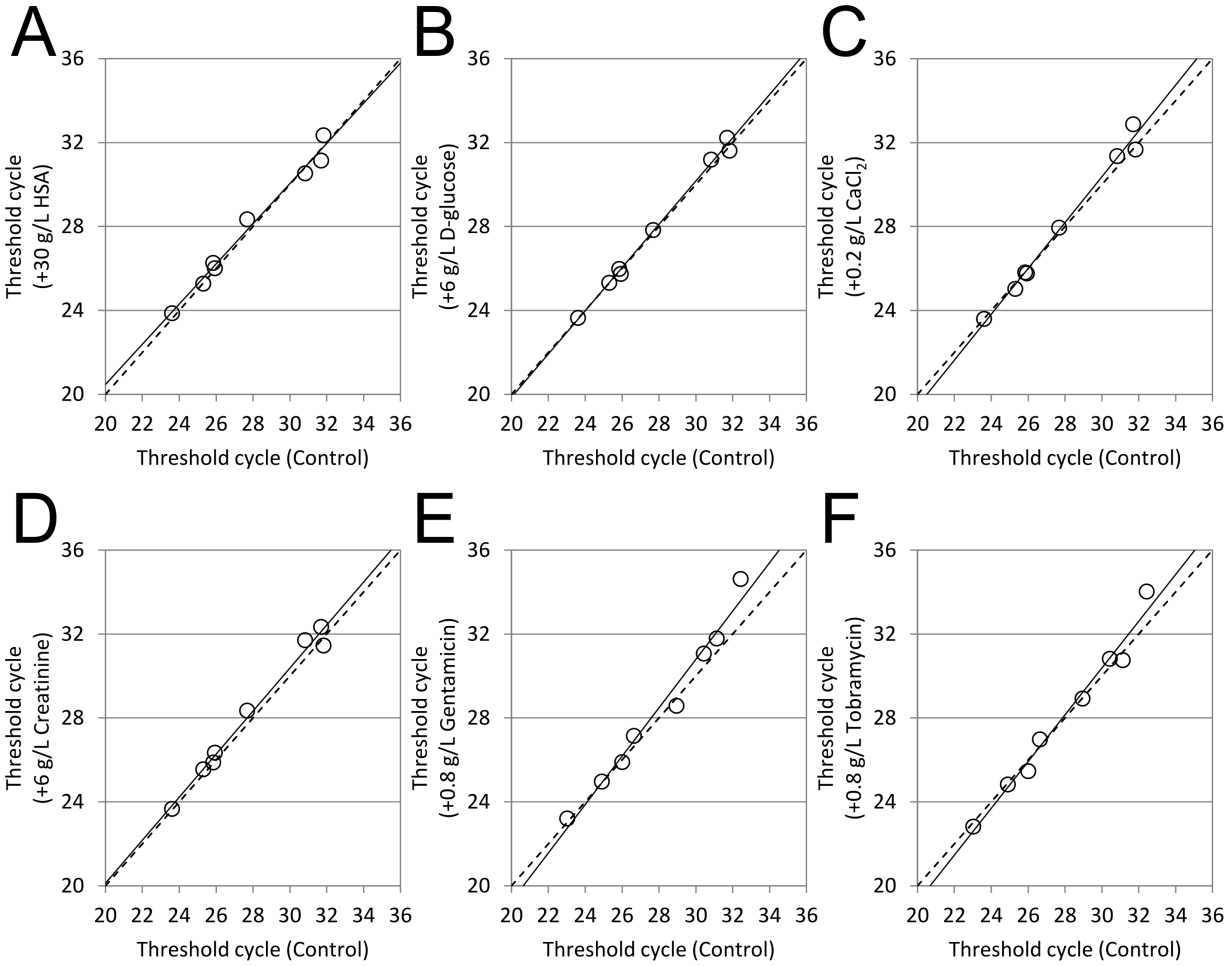
Effect of Urinary Component to EMV mRNA Quantification. Urine sample was obtained from a healthy donor. 30 g/L human serum albumin (A), 6 g/L creatinine (B), 6 g/L D-glucose (C), 0.2 g/L CaCl_2_ (D), 0.8 g/L gentamicin (E) or 0.8 g/L tobramycin (F) was added to the sample. Ten mL urine samples were processed and eight mRNA (*ACTB*, *GAPDH*, *NPHN*, *PDCN*, *SLC12A1*, *UMOD*, *ALB*, *AQP2*) were quantified in triplicate as described in Materials and Methods. Mean Ct values of the chemical supplemented samples were plotted against those of the original sample (control). Solid lines are linear regression curves; their formulas are A. y = 0.958 x+1.307 (R^2^ = 0.984), B. y = 1.025 x−0.383 (R^2^ = 0.985), C. y = 1.031 x−0.753 (R^2^ = 0.995), D. y = 1.093 x−2.405 (R^2^ = 0.988), E. y = 1.152 x−3.782 (R^2^ = 0.975), and F. y = 1.115 x−3.068 (R^2^ = 0.978), respectively. Perforated lines are diagonal lines to indicate perfect matches of RNA profiles.

We further investigated the effects of other urinary components, commonly encountered in clinical settings, such as albumin and creatinine (kidney diseases), glucose (diabetes), calcium (urinary stones), gentamicin and tobramycin (antibiotics for urinary tract infection). The addition of 30 g/L human serum albumin, 6 g/L creatinine, 6 g/L D-glucose, 0.2 g/L CaCl_2_, 0.8 g/L gentamicin or 0.8 g/L tobramycin did not alter the mRNA profile (Figure 4), suggesting that our assay is robust enough to analyze not only healthy urine samples but also pathological ones.

EMV mRNA is our primary interest therefore large urinary sediments such as red blood cells, white blood cells, and casts are removed prior to EMV isolation by low speed centrifugation. EMV mRNA levels were not changed when the different centrifugation speed was applied between 500×g and 2500×g (Figure S3A). By changing the centrifugation speed further, different fractions of EMV could be analyzed. For example, 800×g supernatant may contain exosomes and microvesicles, 10,000×g supernatant may contain exosomes only, and ultracentrifugation (100,000×g) supernatant may not contain EMV except extravesicular mRNA if it exists. *ACTB* and *GAPDH* levels in 10,000×g supernatant were slightly less than in 800×g supernatant, and the difference may account for microvesicle mRNA (Figure S3B) although the mRNA profiles were almost identical (Figure S3C). On the other hand, *ACTB* and *GAPDH* levels in ultracentrifugation supernatant were greatly decreased and its mRNA profile was different from that of 800×g supernatant (Figure S3B, S3D). From the differences of Ct values, it was estimated that ultracentrifugation captured 95 to 97% of urinary EMV, however some abundant genes such as *ACTB*, *GAPDH* and *ALB* were still detected in 100,000×g supernatant, which could be extravesicular mRNA or unprecipitaed EMV. To investigate if extravesicular mRNA can exist in human urine without degradation, rat spleen mRNA was incubated in human whole urine or urine supernatants at 37°C and then quantified. More than 99% of rat spleen mRNA was degraded to within 1 hour (Figure S4). These data further corroborate that our assay is specifically analyzing EMV mRNA.

### Performance Comparison with Standard Ultracentrifugation Method

**Figure 5:**
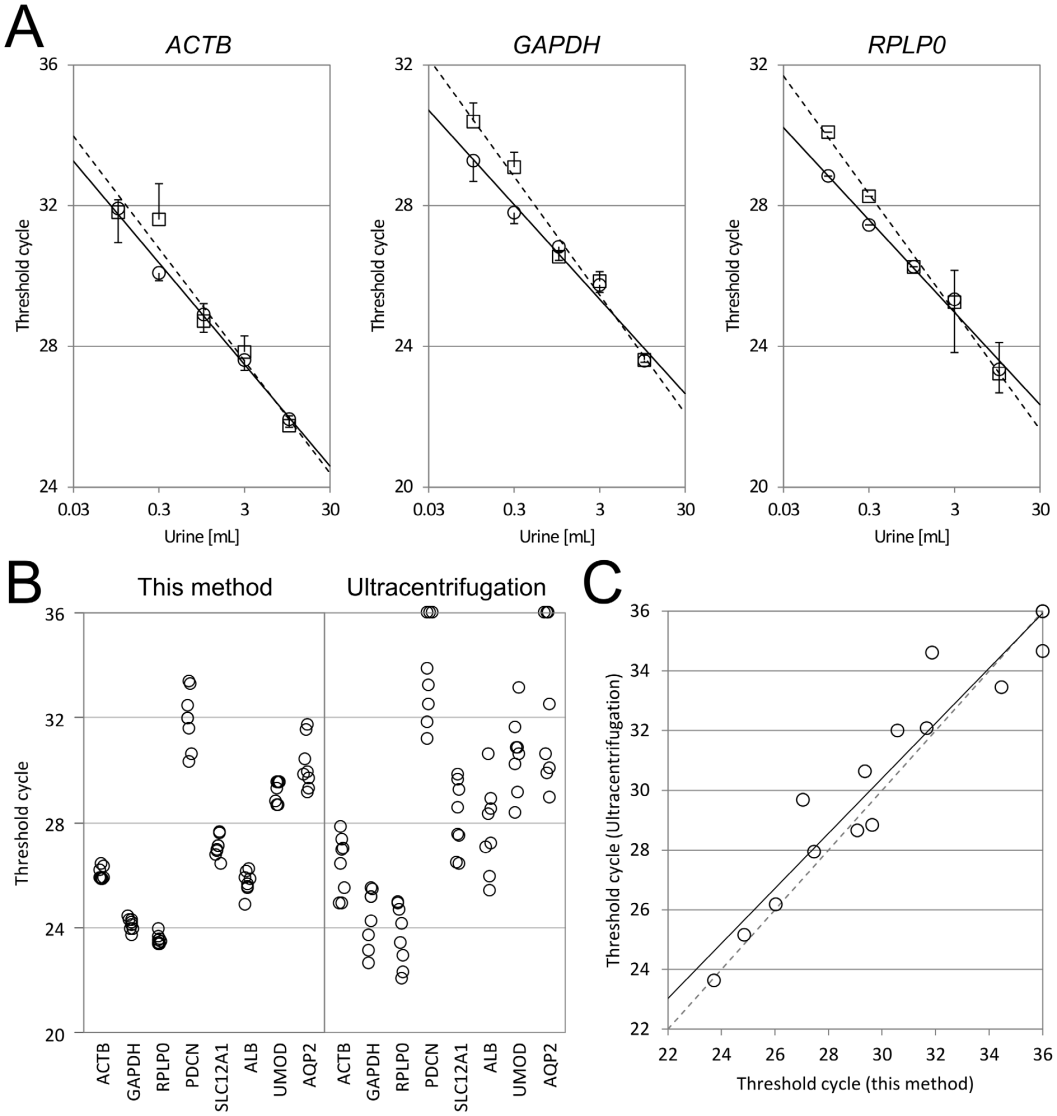
Performance Comparison with Standard Ultracentrifugation Method. A. Assay Linearity Comparison. A spot urine sample was obtained from a healthy donor. One hundred µL to ten mL of the urine sample were processed by EMV capture device (○) or the standard ultracentrifugation method (□) and *ACTB*, *GAPDH* and *RPLP0* were quantified in triplicate as described in Materials and Methods. Mean Ct values were plotted and error bars are standard deviations. Solid and perforated lines are linear regression curves of exosome collection device and the standard ultracentrifugation method, respectively. Their formulas are; [*ACTB*] EMV capture device: y = −2.89 x+28.87 (R^2^ = 0.994) and ultracentrifugation: y = −3.19 x+29.12 (R^2^ = 0.956), [*GAPDH*] EMV capture device: y = −2.69 x+26.63 (R^2^ = 0.980) and ultracentrifugation: y = −3.36 x+27.07 (R^2^ = 0.983), and [*RPLP0*] EMV capture device: y = −2.62 x+26.23 (R^2^ = 0.987) and ultracentrifugation: y = −3.35 x+26.59 (R^2^ = 0.993). B. Intra-assay Reproducibility Comparison. A spot urine sample was obtained from a healthy donor. Eight 10-mL replicates of the urine sample were processed by EMV capture device (left) or the standard ultracentrifugation method (right) and 8 genes (*ACTB*, *GAPDH*, *RPLP0*, *PDCN*, *SLC12A1*, *ALB*, *UMOD*, *AQP2*) were quantified as described in Materials and Methods. Raw Ct values are available in Table S2. C. mRNA Profile Comparison. A spot urine sample was obtained from a healthy donor. Ten mL each of the urine sample was processed by EMV capture device or the standard ultracentrifugation method and 15 genes (*NPHN*, *PDCN*, *SLC12A1*, *ALB*, *UMOD*, *AQP2*, *ACTB*, *GAPDH*, *RPLP0*, *PPIA*, *PGK1*, *B2M*, *HPRT1*, *GUSB1*, *TBP*) were quantified in triplicate as described in Materials and Methods. Mean Ct values of the ultracentrifugation method were plotted against those of EMV capture device. The solid line is linear regression curve and its formula is y = 0.922 x+2.748 (R^2^ = 0.914). The perforated line is a diagonal line to indicate a perfect match of RNA profiles.

The performance of our method was compared with gold standard ultracentrifugation method (100,000×g for 1 hour). For assay linearity, 100 µL to 10 mL of urine samples were processed by our method or the standard method, and three control genes (*ACTB*, *GAPDH*, *RPLP0*) were quantified. Our method showed a wide range of assay linearity between 100 µL and 10 mL of urine samples (Figure 5A). The standard method showed very comparable Ct values to our method between 1 mL and 10 mL of samples, however showed slightly higher Ct values or lower mRNA sensitivities in less than 300 µL samples probably because centrifugation wasn't efficient in smaller volumes of samples. For intra-assay reproducibility, 8 replicates of 10-mL urine samples were processed by both methods and five kidney marker genes and three control genes were quantified (Figure 5B). Mean Ct values were very comparable to each other for all the eight genes, however the coefficients of variation (CV) by our method were below 1.7% for highly expressed genes such as *ACTB*, *GAPDH*, *RPLP0*, *SLC12A1*, *ALB* and *UMOD*, and much less than those by the standard method, whose CV exceeded 4.3% for the same genes (Table S2). For lower abundance gene transcripts such as *PDCN* and *AQP2*, both methods showed higher CV above 3%, however our method was able to detect both genes in 8 out of 8 samples (100% detection rate), while the standard method failed to detect the same genes in 3 out of 8 samples (62.5% detection rate). For RNA profile comparison, from the same urine sample, 15 genes were quantified by both methods, and mean Ct values (N = 3) were plotted against each other (Figure 4C). The coefficient of determination (R^2^) was 0.90, suggesting that both methods showed very similar RNA profiles with each other when mean Ct values were used. These data suggest that our method provided comparable or even superior results to the standard ultracentrifugation method in terms of mRNA assay sensitivity, linearity and intra-assay reproducibility.

### Stability of EMV mRNA during Sample Collection and Storage

The profiles of EMV mRNA were compared between the freshly processed urine samples and the samples incubated at −80°C, 4°C, 20°C or 37°C for 24 hours (Figure S5A–S5D). The profiles of EMV mRNA were consistent during 24-hour incubation, suggesting that the EMV mRNA would be stable up to 24 hours even when stored at room temperature. The profiles of EMV mRNA were also consistent during 10-month storage at −80°C (Figure S5E) or up to two repetitions of freeze-thaw process (Figure S5F–S5H), although more than four repetitions of freeze-thaw process altered the RNA profiles. These data suggest that urinary EMV is stable enough for routine clinical analysis.

### Inter-day and Inter-subject Variations of EMV mRNA in Human Urine Samples

**Figure 6:**
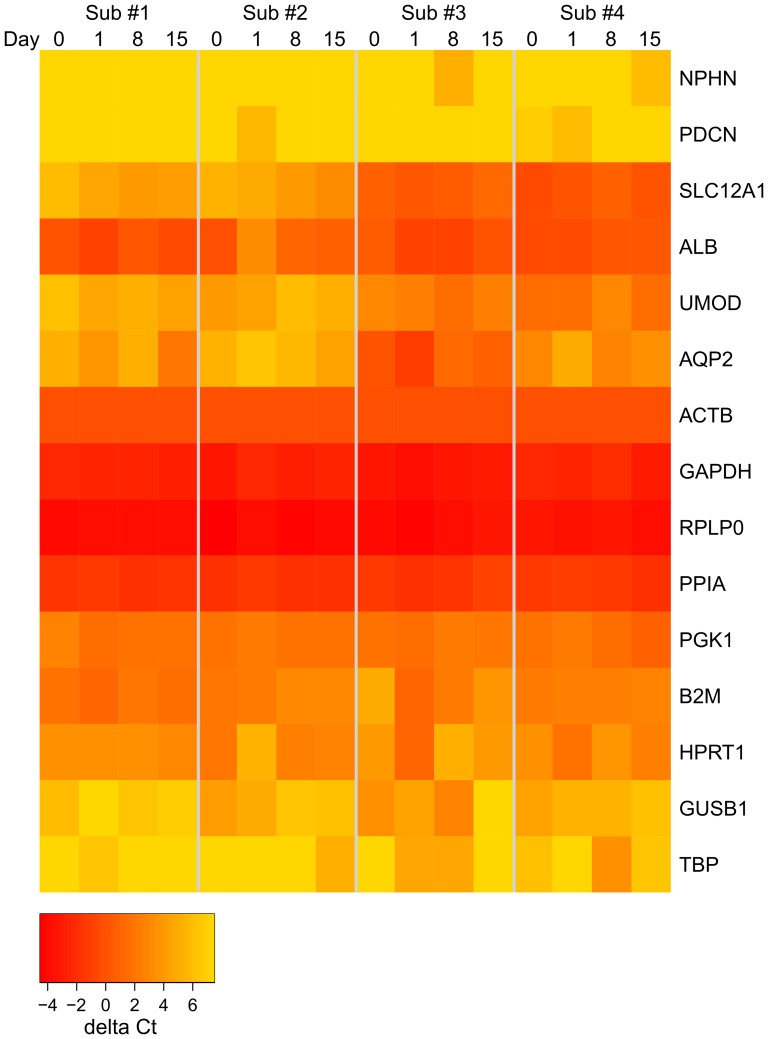
Inter-day and Inter-subject Variation of EMV mRNA in Human Urine. Twelve-hour urine samples (8 pm to 8 am next day) were collected for two weeks (Days 0, 1, 8 and 15) from four healthy subjects. Ten mL urine samples were processed by EMV capture device and 8 genes (*NPHN*, *PDCN*, *SLC12A1*, *ALB*, *UMOD*, *AQP2*, *ACTB*, *GAPDH*, *RPLP0*, *PPIA*, *PGK1*, *B2M*, *HPRT1*, *GUSB1*, *TBP*) were quantified in triplicate and the obtained mean Ct values were normalized by that of *ACTB* and expressed in a heat map format.

Using this assay system, inter-day and inter-subject variations of EMV mRNA were investigated (Figure 6). mRNA data was normalized by the delta Ct method using *ACTB*. Expression levels of five control genes (*ACTB*, *GAPDH*, *RPLP0*, *PPIA* and *PGK1*) were consistent among the subjects through the 2-week study period. The other control genes (*B2M* and *HPRT1*) were expressed less than the above five genes and fluctuated among days and subjects. For kidney-specific genes, the levels of *NPHN* and *PDCN* were very low and occasionally detectable for all 4 subjects (Figure 6). Four kidney specific genes (*SLC12A1*, *UMOD*, *ALB* and *AQP2*) were always detectable and consistent in each individual. However, the expression levels of these four genes varied differently among the four subjects. Especially, the expression levels of *SLC12A1* and *UMOD* were significantly different between Subject 1 and 3, 1 and 4, 2 and 3, 2 and 4 (p<0.0025, non-paired t-test), which may reflect the variation of kidney functions among the subjects (Figure 6).

## Discussion

We developed a unique method to isolate urinary EMV and quantify kidney-specific mRNAs. In comparison to our previous system [12], the new system is useful for processing larger sample volumes without compromising the assay throughput and solved our previous sensitivity issue to process urine samples probably due to the difference in pH and salt concentration, therefore is suitable for urinary EMV analysis. In order to use the method as a platform for biomarker screening and molecular diagnostics for kidney diseases, we validated that the method provided quantitative and reproducible results independent of sample conditions, and comparable or even superior results to the standard ultracentrifugation method. The detection limit of our method is estimated at approximately ten gene copies per 10 mL urine (Figure S6). Additionally, we demonstrated that urinary EMV mRNAs were very stable even after multiple freeze thaw cycles, indicating that the urinary EMV could be an ideal clinical material for research and diagnostics as previously suggested [16]. Moreover, because the standard deviations of Ct values obtained by our method were less than one, it is possible to distinguish as low as two-fold differences of mRNAs among different groups (Figure 5B, Table S2).

Since urinary EMV are released from different sites of the nephron into urine, detailed molecular information of kidney function may be obtained by targeting specific EMV mRNAs. Nephrin (*NPHN*, NCBI Gene ID: 4868) and podocin (*PDCN*, NCBI Gene ID: 7827) are kidney glomerular filtration barrier proteins comprising the slit diaphragm, therefore releasing these EMV mRNA into urine may reflect glomerular filtration functioning such as damages of slit diaphragm of podocytes. *NPHN* and *PDCN* were very low levels in healthy donors' urine (Figure 6), suggesting that the consistent detection or elevation of these genes might be an indicator of glomerular damage. Na-K-Cl cotransporter (*SLC12A1*, NCBI Gene ID: 6557) is a protein involved in the active transport of sodium, potassium, and chloride at the thick ascending limb of the Henle loop and the macula densa, therefore its mRNA could be a useful marker for tubular reabsorption. Uromodulin (*UMOD*, NCBI Gene ID: 7369) is known as Tamm-Horsfall protein, the matrix of the cast, and may act as inhibitor of calcium crystallization and defense against urinary tract infections. As the most abundant urinary protein produced by the thick ascending limb of the Henle loop, detection of *UMOD* mRNA in urine may allow monitoring tubular secretion function. Although urinary albumin is mainly leaked from blood into urine due to abnormal renal glomerulus and used as a kidney failure marker, *ALB* mRNA (NCBI Gene ID: 213) in urinary EMV may be originated from epithelial cells throughout nephrons. Aquaporin 2 (*AQP2*, NCBI Gene ID: 359) reabsorbs water from urine in the collecting duct, therefore *AQP2* may serve as a collecting duct marker. The expression levels of these genes were consistent through the two week study period within the same subject, however significantly different among the subjects (Figure 6), suggesting that these mRNA levels may reflect the kidney functions of the subjects. Indeed, in our previous study, the expression levels of *SLC12A1* and *UMOD* were significantly different between diabetic nephropathy patients and healthy volunteers (presented in American Society of Nephrology in November 2012). Furthermore, the expression levels of the above genes were changed greatly depending on the scores of kidney biopsy for kidney transplantation patients (presented in American Transplant Congress in June 2012). By monitoring the expression levels of these mRNA as well as other kidney specific mRNAs, it may be possible to monitor kidney functions at molecular levels, however further study is still necessary.

In this study, entire EMV fractions were isolated and poly(A)^+^ RNA were purified. Thus, we do not know the origin, size, and shape of the EMV in this analysis. However, these disadvantages were overcome by the detection of site- and function-specific mRNAs. Moreover, by designing appropriate primers in each gene, mutations and splicing variants can be analyzed. The RNAs purified in this assay are those with intact 3′ poly(A)^+^ tails, full length and degraded mRNA can be differentiated by primers targeting the 5′ and 3′ end regions, respectively. Moreover, all species of mRNA can be quantified by using RNA-seq (unpublished data).

One of the major problems is the expression of the results. In many mRNA studies, the results of target gene are normalized by the values of control gene. We also expressed the mRNA expression profiles by the prevalent delta Ct method. This is applicable only when the amount of control gene represents control status. At least, it was confirmed that the five conventional control genes (*ACTB*, *GAPDH*, *RPLP0*, *PPIA* and *PGK1*) are expressed abundantly and consistent among subjects for two weeks. However, in urinary EMV, alteration of the levels of control genes itself may indicate pathological conditions. Thus, a conventional normalization procedure may not be applicable. As demonstrated in our previous study, mRNA purification yield and cDNA synthesis efficiency are consistent regardless of the expression levels, length, and the sequences of mRNA (approximately 10%) [14]. Moreover, as shown in this study, EMV capture efficiency is also consistent more than 95%. Thus, the levels of each gene in each sample were successfully quantified as gene copy number/mg creatinine as shown in Figure S6. Such quantification of EMV mRNA in urine may be useful to monitor the clinical courses as well as the conventional urinary markers.

In conclusion, we present a unique method for EMV isolation and mRNA quantification in human urine. Although detection of microRNA and proteins in urinary EMV still need further protocol optimization, analytical validation for mRNA detection was completed, and will be readily applicable to biomarker discovery projects and the development of molecular diagnostics for kidney diseases.

## Supporting Information

Figure S1 **Scanning electron microscope (SEM) analysis of EMV capture material.** Human urine was applied to exosome collection tube (Hitachi Chemical Research Center (HCR), Irvine, CA) and centrifuged at 2,000×g for 5 min. The filter membrane was removed, dried, sputter-coated, and analyzed by SEM (S-4800, Hitachi High-Technologies, Tokyo, Japan).
(PDF)

Figure S2 **EMV Capture Yield.** EMV capture yield was calculated by comparing EMV concentrations before and after filtration. Isolated urinary EMV in 1× PBS was applied to the EMV filter material. The applied EMV and filtrate were lysed by adding equal volumes of 2× Lysis buffer, and *ACTB* (○) and *GAPDH* (□) were quantified in triplicate as described in Materials and Methods. Mean Ct values were plotted. Error bars are standard deviations. From the obtained Ct values, exosome recovery yields were 99.6% for *ACTB* and 99.5% for *GAPDH* using the following formulation: [Recovery yield] = 100−100×2^([mean Ct of applied EMV]−[mean Ct of filtrate])^×100.
(PDF)

Figure S3 **mRNA quantity and profile comparison among different EMV fractions.** A. Urine sample was obtained from a healthy donor. Aliquoted sample was centrifuged at 500, 800, 1500 or 2450×g for 15 min, and the supernatants were collected. *ACTB* (○) and *GAPDH* (□) in 10 mL urine supernatants were quantified in triplicate as described in Materials and Methods. Mean Ct values of the incubated samples were plotted. Error bars are standard deviations. B–D. Urine sample was obtained from a healthy donor and centrifuged at 800×g for 15 min. The supernatant was collected and centrifuged at 10,000×g for 30 min. The supernatant was collected and centrifuged at 100,000×g for 1 hour. Eight mRNA (*ACTB*, *GAPDH*, *NPHN*, *PDCN*, *SLC12A1*, *UMOD*, *ALB*, *AQP2*) in 10 mL supernatants at each centrifugation step were quantified in triplicate as described in Materials and Methods. B. Mean Ct values of *ACTB* (○) and *GAPDH* (□) were plotted and error bars are standard deviations. C and D. Mean Ct values of 10,000×g (C) or 100,000×g supernatant (D) were plotted against those of 800×g supernatant. Solid lines are linear regression curves and perforated lines are diagonal lines to indicate perfect matches of RNA profiles.
(PDF)

Figure S4 **Ribonuclease activity in human urine.** Ribonuclease activity of human urine samples was investigated. Two ng rat spleen mRNA was suspended in 20 µL PBS (#1), human whole urine (#2), 800×g supernatant (#3) or 100,000×g supernatant (#4), and incubated at 37°C for 1 hour. For positive control, 2 ng rat spleen mRNA was spiked in 20 µL PBS and incubated on ice for 1 hour. For negative control, whole urine was incubated at 37°C for 1 hour without rat spleen mRNA. Following the incubation, the samples were lysed by adding 180 µL Lysis buffer and incubating at 37°C for 10 min. 60 µL each of lysates was transferred to oligo(dT) immobilized microplate for mRNA isolation and quantification in triplicate as described in Materials and Methods. Rat *Actb* (○) and *Gapdh* (□) were quantified.
(PDF)

Figure S5 **Stability of Urinary EMV mRNA.** Urine samples were obtained from healthy donors and aliquot to 40 mL each. Fresh samples were processed immediately after sample collection and the rest of the samples were incubated at several conditions: 24 hours at −80°C and thawed at 37°C for 15 min (A), 24 hours at 4°C (B), 24 hours at 20°C (C), 24 hours at 37°C (D), 10 months at −80°C and thawed at 37°C for 15 min (E) or 24 hours at −80°C, followed by two repeats (F), four repeats (G) or eight repeats (H) of freeze-thaw cycle (frozen at −80°C at least for 2 hours and thawed at 37°C for 15 min). The samples were centrifuged at 800×g for 15 min and 10 mL each was processed as described in Materials and Methods. Eight mRNA (*ACTB*, *GAPDH*, *NPHN*, *PDCN*, *SLC12A1*, *UMOD*, *ALB*, *AQP2*) were quantified in triplicate and mean Ct values of the incubated samples were plotted against those of the fresh sample. Solid lines are linear regression curves and perforated lines are diagonal lines to indicate perfect matches of RNA profiles.
(PDF)

Figure S6 **Intra-day and Intra-subject Variation of EMV mRNA in Human Urine.** The data in Figure 6 were expressed in gene copy number per urinary creatinine as described in Materials and Methods. Urinary creatinine concentration was measured by creatinine assay kit (Oxford Biomedical Research, Rochester Hills, MI). Intra-day expression levels of the eight genes are shown: Subjects #1 (○), #2 (□), #3 (Δ) and #4 (◊).
(PDF)

Table S1 **Primer sequences.**
(PDF)

Table S2 **Comparison of intra-assay reproducibility.**
(PDF)
