# Supplementary figures and images for: Development of Glomerulus-, Tubule-, and Collecting Duct-Specific mRNA Assay in Human Urinary Exosomes and Microvesicles
Source: PLoS One. 2014 Oct 2;9(10):e109074. doi: 10.1371/journal.pone.0109074 (PMC4183527; doi:10.1371/journal.pone.0109074)

**Figure S1. Scanning electron microscope (SEM) analysis of EMV capture material**

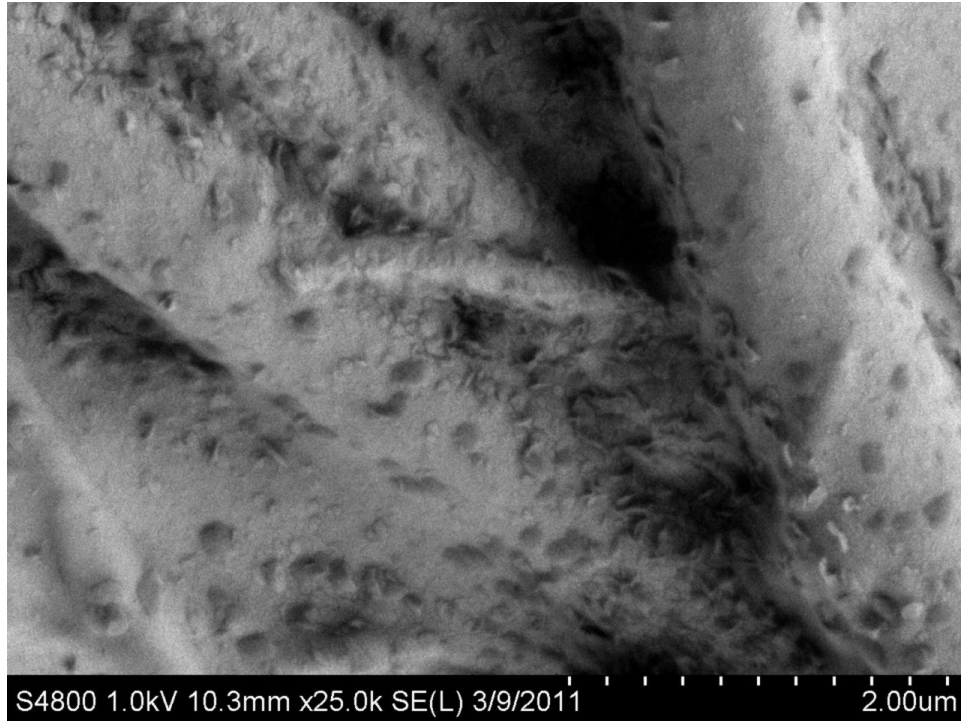

Supplement: Figure S1 — Scanning electron microscope (SEM) analysis of EMV capture material. Human urine was applied to exosome collection tube (Hitachi Chemical Research Center (HCR), Irvine, CA) and centrifuged at 2,000×g for 5 min. The filter membrane was removed, dried, sputter-coated, and analyzed by SEM (S-4800, Hitachi High-Technologies, Tokyo, Japan). (PDF) [file pone.0109074.s001.pdf]

**Figure S2. EMV Capture Yield**

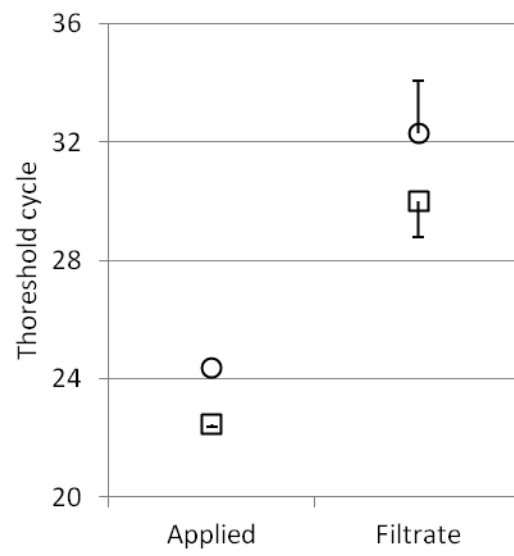

Supplement: Figure S2 — EMV Capture Yield. EMV capture yield was calculated by comparing EMV concentrations before and after filtration. Isolated urinary EMV in 1× PBS was applied to the EMV filter material. The applied EMV and filtrate were lysed by adding equal volumes of 2× Lysis buffer, and ACTB (○) and GAPDH (□) were quantified in triplicate as described in Materials and Methods. Mean Ct values were plotted. Error bars are standard deviations. From the obtained Ct values, exosome recovery yields were 99.6% for ACTB and 99.5% for GAPDH using the following formulation: [Recovery yield] = 100−100×2([mean Ct of applied EMV]−[mean Ct of filtrate])×100. (PDF) [file pone.0109074.s002.pdf]

Figure S3. mRNA quantity and profile comparison among different EMV fractions

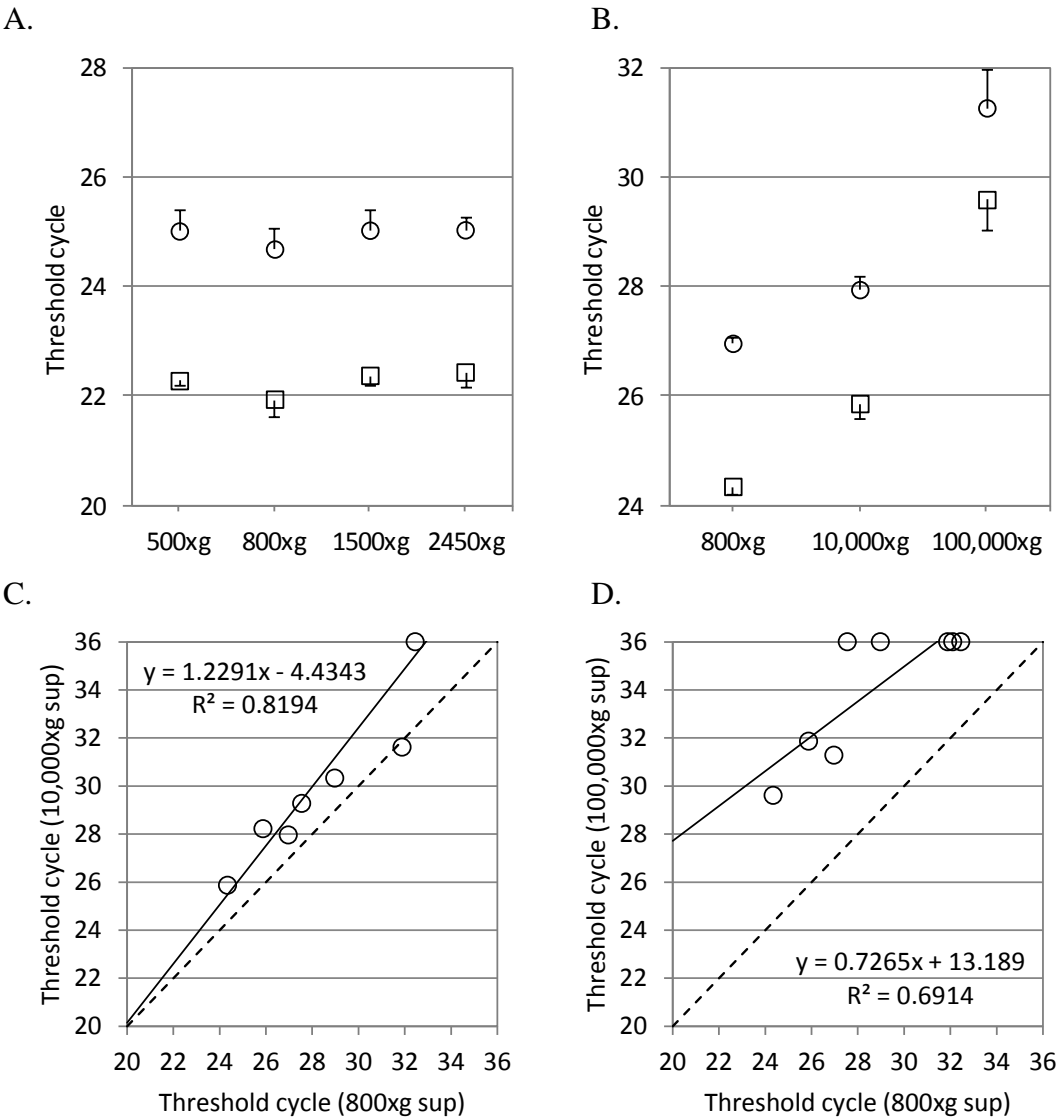

Supplement: Figure S3 — mRNA quantity and profile comparison among different EMV fractions. A. Urine sample was obtained from a healthy donor. Aliquoted sample was centrifuged at 500, 800, 1500 or 2450×g for 15 min, and the supernatants were collected. ACTB (○) and GAPDH (□) in 10 mL urine supernatants were quantified in triplicate as described in Materials and Methods. Mean Ct values of the incubated samples were plotted. Error bars are standard deviations. B–D. Urine sample was obtained from a healthy donor and centrifuged at 800×g for 15 min. The supernatant was collected and centrifuged at 10,000×g for 30 min. The supernatant was collected and centrifuged at 100,000×g for 1 hour. Eight mRNA (ACTB, GAPDH, NPHN, PDCN, SLC12A1, UMOD, ALB, AQP2) in 10 mL supernatants at each centrifugation step were quantified in triplicate as described in Materials and Methods. B. Mean Ct values of ACTB (○) and GAPDH (□) were plotted and error bars are standard deviations. C and D. Mean Ct values of 10,000×g (C) or 100,000×g supernatant (D) were plotted against those of 800×g supernatant. Solid lines are linear regression curves and perforated lines are diagonal lines to indicate perfect matches of RNA profiles. (PDF) [file pone.0109074.s003.pdf]

**Figure S4. Ribonuclease activity in human urine**

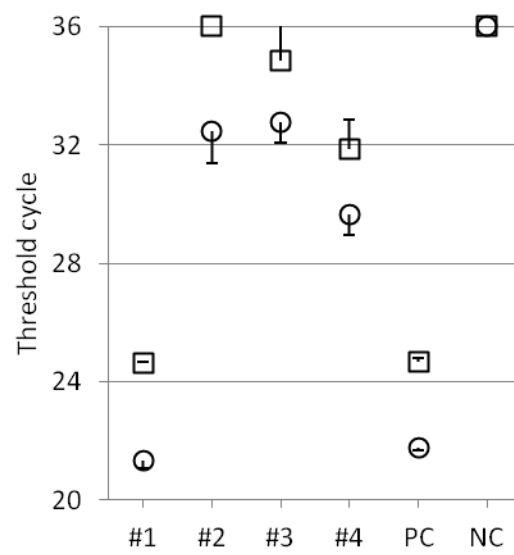

Supplement: Figure S4 — Ribonuclease activity in human urine. Ribonuclease activity of human urine samples was investigated. Two ng rat spleen mRNA was suspended in 20 µL PBS (#1), human whole urine (#2), 800×g supernatant (#3) or 100,000×g supernatant (#4), and incubated at 37°C for 1 hour. For positive control, 2 ng rat spleen mRNA was spiked in 20 µL PBS and incubated on ice for 1 hour. For negative control, whole urine was incubated at 37°C for 1 hour without rat spleen mRNA. Following the incubation, the samples were lysed by adding 180 µL Lysis buffer and incubating at 37°C for 10 min. 60 µL each of lysates was transferred to oligo(dT) immobilized microplate for mRNA isolation and quantification in triplicate as described in Materials and Methods. Rat Actb (○) and Gapdh (□) were quantified. (PDF) [file pone.0109074.s004.pdf]

**Figure S5. Stability of Urinary EMV mRNA.**

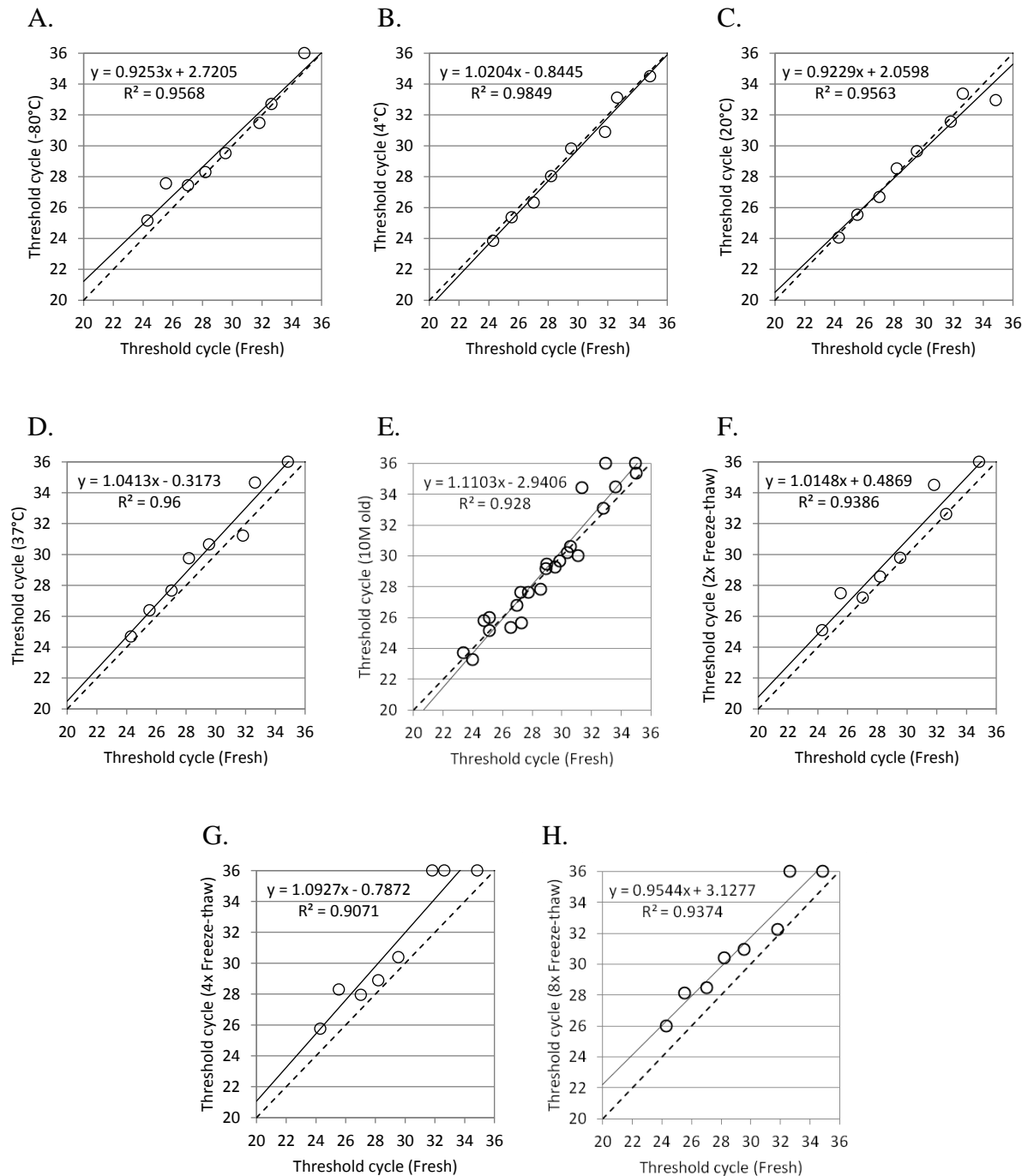

Supplement: Figure S5 — Stability of Urinary EMV mRNA. Urine samples were obtained from healthy donors and aliquot to 40 mL each. Fresh samples were processed immediately after sample collection and the rest of the samples were incubated at several conditions: 24 hours at −80°C and thawed at 37°C for 15 min (A), 24 hours at 4°C (B), 24 hours at 20°C (C), 24 hours at 37°C (D), 10 months at −80°C and thawed at 37°C for 15 min (E) or 24 hours at −80°C, followed by two repeats (F), four repeats (G) or eight repeats (H) of freeze-thaw cycle (frozen at −80°C at least for 2 hours and thawed at 37°C for 15 min). The samples were centrifuged at 800×g for 15 min and 10 mL each was processed as described in Materials and Methods. Eight mRNA (ACTB, GAPDH, NPHN, PDCN, SLC12A1, UMOD, ALB, AQP2) were quantified in triplicate and mean Ct values of the incubated samples were plotted against those of the fresh sample. Solid lines are linear regression curves and perforated lines are diagonal lines to indicate perfect matches of RNA profiles. (PDF) [file pone.0109074.s005.pdf]

**Figure S6. Intra-day and Intra-subject Variation of EMV mRNA in Human Urine.**

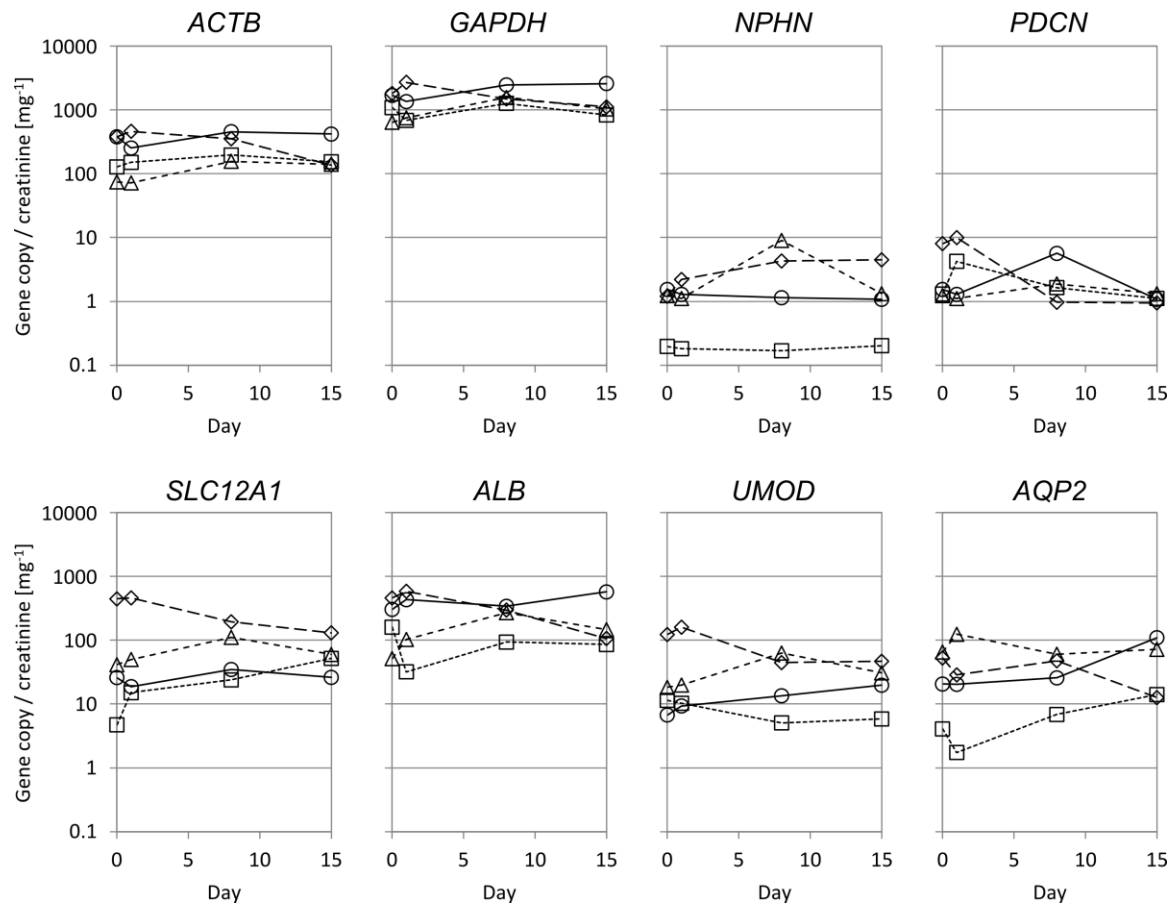

Supplement: Figure S6 — Intra-day and Intra-subject Variation of EMV mRNA in Human Urine. The data in Figure 6 were expressed in gene copy number per urinary creatinine as described in Materials and Methods. Urinary creatinine concentration was measured by creatinine assay kit (Oxford Biomedical Research, Rochester Hills, MI). Intra-day expression levels of the eight genes are shown: Subjects #1 (○), #2 (□), #3 (Δ) and #4 (◊). (PDF) [file pone.0109074.s006.pdf]
