# Supplementary material for: Development of Glomerulus-, Tubule-, and Collecting Duct-Specific mRNA Assay in Human Urinary Exosomes and Microvesicles
Source: PLoS One. 2014 Oct 2;9(10):e109074. doi: 10.1371/journal.pone.0109074 (PMC4183527; doi:10.1371/journal.pone.0109074)
Supplement: Table S1 — Primer sequences. (PDF) [file pone.0109074.s007.pdf]

Table S1. Primer sequences

Gene	Sense (5' to 3')	Anti-sense (5' to 3')
NPHN	CTTCCCTGGGCACTTGTATGA	TCATAGATTCCTCTTGGATCCTGAT
PDCN	AGGATGGCAGCTGAGATTCTGT	AGAGACTGAAGGGTGTGGAGGTAT
SLC12A1	ACTCCAGAGCTGCTAATCTCATTGT	AACTAGTAAGACAGGTGGGAGGTTCT
ALB	TGCAAGGCTGACGATAAGGA	GTAGGCTGAGATGCTTTTAAATGTGA
UMOD	CCTGAACTTGGGTCCCATCA	GCCCCAAGCTGCTAAAAGC
AQP2	CAATGCCTCCCAGCTAGACTGT	TGCTTATTATATGCAGAGCAGTTGAA
ACTB	CCTGGCACCCAGCACAAT	GCCGATCCACACGGAGTACT
GAPDH	CCCACTCCTCCACCTTTGAC	CATACCAGGAAATGAGCTTGACAA
RPLP0	TGCATCAGTACCCCATTCTATCA	GGTGTAATCCGTCTCCACAGACA
Actb (Rat)	TCTGTGTGGATTGGTGGCTCTA	CTGCTTGCTGATCCACATCTG
Gapdh (Rat)	ACCAGGTTGTCTCCTGTGACTTC	CAGGAAATGAGCTTCACAAAGTTG
