# Supplementary material for: Development of Glomerulus-, Tubule-, and Collecting Duct-Specific mRNA Assay in Human Urinary Exosomes and Microvesicles
Source: PLoS One. 2014 Oct 2;9(10):e109074. doi: 10.1371/journal.pone.0109074 (PMC4183527; doi:10.1371/journal.pone.0109074)
Supplement: Table S2 — Comparison of intra-assay reproducibility. (PDF) [file pone.0109074.s008.pdf]

Table S2. Comparison of intra-assay reproducibility

		#1	#2	#3	#4	#5	#6	#7	#8	Mean Ct	SD	CV
ACTB	This method	26.2	26.3	25.9	25.9	26.4	25.9	25.9	25.9	26.0	0.2	0.9%
	Standard method	27.3	24.9	27.0	24.9	26.4	27.0	25.5	27.9	26.4	1.1	4.3%
GAPDH	This method	23.9	24.4	24.2	23.7	24.3	24.1	24.3	23.9	24.1	0.2	1.0%
	Standard method	25.5	23.1	25.5	22.7	24.2	25.2	23.7	25.2	24.4	1.1	4.6%
RPLP0	This method	23.4	23.6	23.6	23.4	23.5	23.4	23.9	23.4	23.5	0.2	0.8%
	Standard method	24.9	22.3	25.0	22.1	23.4	24.1	22.9	24.7	23.7	1.2	4.9%
PDCN	This method	30.3	32.0	33.3	31.9	33.4	30.6	32.4	31.6	31.9	1.1	3.5%
	Standard method	33.9	31.2	N.D.	31.8	32.5	N.D.	33.2	N.D.	32.5	1.1	3.3%
SLC12A1	This method	27.1	27.7	26.4	26.9	27.1	26.8	27.6	26.9	27.1	0.4	1.5%
	Standard method	29.8	26.5	28.6	26.4	27.5	29.2	27.5	29.6	28.1	1.4	4.8%
ALB	This method	25.9	26.2	24.9	25.5	25.9	25.7	26.2	25.5	25.7	0.4	1.7%
	Standard method	28.9	25.9	30.6	25.4	27.1	28.5	27.2	28.3	27.7	1.7	6.1%
UMOD	This method	29.3	29.5	28.8	29.5	28.6	28.7	29.6	29.6	29.2	0.4	1.4%
	Standard method	30.8	28.4	33.1	29.1	30.2	30.6	30.9	31.6	30.6	1.5	4.8%
AQP2	This method	30.4	31.7	29.8	29.3	31.5	29.7	29.1	29.9	30.2	1.0	3.2%
	Standard method	N.D.	30.1	32.5	29.0	30.6	N.D.	29.9	N.D.	30.4	1.3	4.3%
